# PHD Finger Protein 19 Promotes Cardiac Hypertrophy via Epigenetically Regulating SIRT2

**DOI:** 10.1007/s12012-021-09639-0

**Published:** 2021-02-21

**Authors:** Wei Gu, Yutong Cheng, Su Wang, Tao Sun, Zhizhong Li

**Affiliations:** Department of Cardiology, Beijing Anzhen Hospital, Capital Medical University and Beijing Institute of Heart Lung and Blood Vessel Diseases, 2 Anzhen Road, Beijing, China

**Keywords:** Cardiac hypertrophy, Epigenetics, PHF19, Histone methylation, SIRT2

## Abstract

Epigenetic regulations essentially participate in the development of cardiomyocyte hypertrophy. PHD finger protein 19 (PHF19) is a polycomb protein that controls H3K36me3 and H3K27me3. However, the roles of PHF19 in cardiac hypertrophy remain unknown. Here in this work, we observed that PHF19 promoted cardiac hypertrophy via epigenetically targeting SIRT2. In angiotensin II (Ang II)-induced cardiomyocyte hypertrophy, adenovirus-mediated knockdown of *Phf19* reduced the increase in cardiomyocyte size, repressed the expression of hypertrophic marker genes *Anp* and *Bnp*, as well as inhibited protein synthesis. By contrast, *Phf19* overexpression promoted Ang II-induced cardiomyocyte hypertrophy in vitro. We also knocked down *Phf19* expression in mouse hearts in vivo. The results demonstrated that *Phf19* knockdown reduced Ang II-induced decline in cardiac fraction shortening and ejection fraction. *Phf19* knockdown also inhibited Ang II-mediated increase in heart weight, reduced cardiomyocyte size, and repressed the expression of hypertrophic marker genes in mouse hearts. Further mechanism studies showed that PHF19 suppressed the expression of SIRT2, which contributed to the function of PHF19 during cardiomyocyte hypertrophy. PHF19 bound the promoter of SIRT2 and regulated the balance between H3K27me3 and H3K36me3 to repress the expression of SIRT2 in vitro and in vivo. In human hypertrophic hearts, the overexpression of PHF19 and downregulation of SIRT2 were observed. Of importance, *PHF19* expression was positively correlated with hypertrophic marker genes *ANP* and *BNP* but negatively correlated with *SIRT2* in human hypertrophic hearts. Therefore, our findings demonstrated that PHF19 promoted the development of cardiac hypertrophy via epigenetically regulating SIRT2.

**Supplementary Information:**

The online version of this article (10.1007/s12012-021-09639-0) contains supplementary material, which is available to authorized users.

## Introduction

Cardiovascular diseases have become the first leading cause of death worldwide [1–4]. Various cardiovascular risk factors can lead to cardiac hypertrophy, one of the fundamental mechanisms underlying multiple heart diseases. In general, hypertension, myocardial infarction, and neuroendocrine factors can lead to cardiac hypertrophy. Cardiac hypertrophy is classified as physiological and pathological hypertrophy. Physiological hypertrophy of the hearts occurs during development or pregnancy, and this type of hypertrophy is revisable [5]. Under pathological conditions, the cardiomyocytes cannot proliferate to support the decreased cardiomyocyte numbers or increased demand for overload. Instead, the cardiomyocytes undergo pathologically hypertrophic growth. However, this pathological hypertrophy is irreversible and can finally result in heart failure and arrhythmia [6–8]. The understanding of the mechanisms underlying pathological hypertrophy is critical for the treatment of cardiac diseases.

During the past decade, accumulating studies have demonstrated the critical roles of epigenetic regulators in cardiac hypertrophy. Histone acetylation and methylation are critically involved in the development of cardiac hypertrophy [5]. For instance, Jumonji domain-containing 2A (JMJD2A) promoted the development of pathological cardiac hypertrophy via regulating H3K9me3 and expression of four-and-a-half LIM domains 1 [9]. The histone demethylase JMJD1C was also reported to modulate CAMKK2-AMPK signaling to contribute to cardiac hypertrophy [10]. H3K9me2-specific demethylase KDM3A promoted cardiac hypertrophy and fibrosis in response to pressure-overload. Cardiomyocyte KDM3A activated Timp1 transcription to promote cardiac remodeling [11]. Histone acetylation was also reported to participate in cardiac hypertrophy. Class I histone deacetylase (HDAC) was demonstrated to promote cardiac hypertrophy, and inhibitors of class I HDACs are potential drugs for the treatment of cardiac hypertrophy [6, 12].

PHD finger protein 19 (PHF19) is a polycomb protein that controls H3K36me3 and H3K27me3. For instance, PHF19 bound H3K36me3 to recruit PRC2 and demethylase NO66 to embryonic stem cell genes during differentiation [13]. PHF19 also controlled hematopoietic stem cell state and differentiation [14]. In multiple myeloma, PHF19 promoted tumorigenicity through PRC2 activation and broad H3K27me3 domain formation [15, 16]. Besides, PHF19 also regulated the proliferation and invasiveness of prostate cancer cells via H3K27me3 deposition [17]. However, the roles of PHF19 in the cardiovascular system remain unknown.

SIRT2 belongs to the class III histone deacetylase Sirtuin family. SIRT2 can locate in the cytoplasm, nucleus, and mitochondria and controls the deacetylation of histones, cytoplasmic, and mitochondrial proteins [18]. Several studies have reported the critical roles of SIRT2 in pathological hypertrophy [19–21]. For instance, Tang et al*.* reported that SIRT2 repressed the development of aging-related cardiac hypertrophy via deacetylating LKB1 and activating the LKB1-AMPK signaling pathway [19]. However, the upstream regulators of SIRT2 expression during cardiac hypertrophy remain unknown.

In the present study, we identified that PHF19 promoted cardiac hypertrophy via downregulating SIRT2. The expression of PHF19 was upregulated in hypertrophic hearts. Overexpression of PHF19 promoted cardiomyocyte hypertrophy in vitro and in vivo*.* Mechanism study revealed that PHF19 epigenetically repressed the expression of SIRT2 via controlling H3K36me3 and H3K27me3 switch. Finally, we observed that SIRT2 was critically involved in PHF19 function in cardiomyocyte hypertrophy.

## Materials and Methods

### Human Heart Samples

All human heart samples were collected at Beijing Anzhen Hospital, Capital Medical University, from 2016 to 2020. Before use, the samples were collected and stored at − 80 °C. The study was approved by the Ethics Committee of Beijing Anzhen Hospital, and every patient has signed the informed consent.

### Animal Models

Cardiac hypertrophy in 8–12-week-old male C57BL/6 mice were induced by chronic infusion of angiotensin II (Sigma, A9525, 1.5 mg/kg/day) with Alzet 2004 minipump for 4 weeks. The ejection fraction and fraction shortening were monitored by echocardiography. Heart rate and blood pressure were also measured at the end of the experiment. Heart weight, body weight, and tibia length were also analyzed. The animal study was approved by the Animal Study Ethics Committee of Beijing Anzhen Hospital.

### Isolation of Neonatal Rat Cardiomyocytes

Primary cardiomyocytes were isolated from neonatal (1–3 day) Sprague Dawley rat left ventricles, as described previously [22]. The neonatal rat cardiomyocytes were cultured in DMEM (Gibco) supplemented with 10% FBS (Gibco), in the presence of BrdU (Sigma), at the condition of 37 °C and 5% CO_2_.

### Adenovirus and Adeno-Associated Virus

The adenovirus system was used for overexpression or knockdown of *Phf19* in rat cardiomyocytes. In brief, sh*Phf19* (5′-GGAAAGAGCAAGCCTGGTTTG-3′), *Phf19*-overexpressing, and respective control constructs were cloned into adenovirus-packaging plasmids, and the adenovirus was packaged in HEK293A cells, as described previously [23]. For the knockdown of *Phf19* in mouse hearts, an adeno-associated virus 9 (AAV9) system was applied, as described previously [24]. AAV9 expressing sh*Phf19* (5′-GCTTGCACACAGTGCCTTAGT-3′) or control shRNA was obtained and injected into neonatal mice. The neonatal mice received different AAV9 (3 × 10^12^ vg per mouse) via systemic administration into the temporal vein using a 30-G needle. Each mouse received a single dose of AAV9-shRNA. Eight weeks later, the mice were subjected to cardiac hypertrophy induction with Ang II.

### In Vitro Model of Cardiomyocyte Hypertrophy

For cardiomyocyte hypertrophy in vitro, cardiomyocytes were pretreated with 1% FBS overnight and then treated with Ang II (1 μM) for 48 h. The cardiomyocytes were stained with a-actinin antibody (Sigma), and cardiomyocyte size of 50 cells per group was analyzed with Image J via a blinded fashion. The average cardiomyocyte sizes of three independent experiments were used for analysis. For inhibition of SIRT2, the cells were treated with SIRT2 inhibitor AGK2 (10 μM) or DMSO.

### Quantitative Real-Time PCR

For quantitative real-time PCR, the SYBR Green master mix kit (ThermoFisher) was applied. Briefly, total RNA was obtained from cells or tissues with TRIzol reagent (Invitrogen), and the first-strand cDNA was generated with the cDNA Synthesis Kit (Sigma), followed by qRT-PCR. The primers used in this study are listed in Table 1.

**Table 1:** Primers used in this study

Gene symbol	Forward primer (5′-3′)	Reverse primer (5′-3′)
Human *ANP*	AGCGGACTGGGCTGTAACAG	ACGACGCCAGCATGAGCTCCTTC
Human *BNP*	TGGAAACGTCCGGGTTACAG	CTGATCCGGTCCATCTTCCT
Human *PHF19*	ACTCGGGACTCCTATGGTGC	CCTCCGTCAGTTTGGACATCA
Human *SIRT2*	TGCGGAACTTATTCTCCCAGA	GAGAGCGAAAGTCGGGGAT
Human *GAPDH*	GGAGCGAGATCCCTCCAAAAT	GGCTGTTGTCATACTTCTCATGG
Mouse *Anp*	GTGCGGTGTCCAACACAGAT	TCCAATCCTGTCAATCCTAC
Mouse *Bnp*	GAGGTCACTCCTATCCTCTGG	GCCATTTCCTCCGACTTTTCT
Mouse *Phf19*	GGCCTGGGTTACCACCAAC	CGTCGGCAGAACCAATGAG
Mouse *Sirt2*	GCGGGTATCCCTGACTTCC	CGTGTCTATGTTCTGCGTGTAG
Mouse *Gapdh*	TGGATTTGGACGCATTGGTC	TTTGCACTGGTACGTGTTGAT
Rat *Anp*	GCTTCCAGGCCATATTGGAG	GCTTCCAGGCCATATTGGAG
Rat *Bnp*	GAGGTCACTCCTATCCTCTGG	GCCATTTCCTCCGACTTTTCT
Rat *Phf19*	TCTGCCGACGCTGCATTTT	CAGCACCATTTTCACTGCCTG
Rat *Sirt2*	CCACGGCACCTTCTACACATC	CACCTGGGAGTTGCTTCTGAG
Rat *Gapdh*	AGGTCGGTGTGAACGGATTTG	TGTAGACCATGTAGTTGAGGT

### Western Blot

Heart tissues and cardiomyocytes were subjected to protein isolation with RIPA reagent. The protein samples were treated with protease inhibitors to reduce degeneration. 40 ug of total protein was subjected to western blot with the protocol described previously [25]. The following antibodies were used: anti-PHF19 antibody (Cell Signaling Technology), anti-SIRT2 antibody (Proteintech), anti-GAPDH (Protein tech).  

### Chromatin Immunoprecipitation

To analyze the enrichment of proteins at SIRT2 promoter, chromatin immunoprecipitation (ChIP) was performed with IgG, PHF19, H3K36me3, H3K27me3 antibodies using the Pierce™ Agarose ChIP Kit (ThermoFisher). The antibodies for ChIP assay were purchased from Abcam. The relative enrichment of each protein was analyzed by qRT-PCR and normalized by Input.

### Protein Synthesis Assay

To analyze protein synthesis, the incorporation of [^3^H]-leucine was monitored as described previously [21]. The cells were treated as described in figure legends, and [^3^H]-leucine was added two hours before testing.

### Histochemical Analysis

For histochemical analysis, the H&E staining was performed with the kit. Briefly, heart tissues were fixed in 4% paraformaldehyde overnight and then embedded in paraffin. The tissues were cut into 5 um sections. The sections were applied to H&E staining with the H&E Staining Kit (Abcam) according to the manual. Cardiomyocyte size was quantified with Image J.

### Statistical Analysis

All experiments were repeated at least three times, and the values are shown as mean ± SD. For the analysis of two group data, Student’s *t* test was applied. For analysis of more than two groups, one-way ANOVA followed by Tukey post hoc test was applied. All statistical analysis was performed with GraphPad Prism 7.

## Results

### PHF19 Promotes Cardiomyocyte Hypertrophy In Vitro

**Fig. 1:**
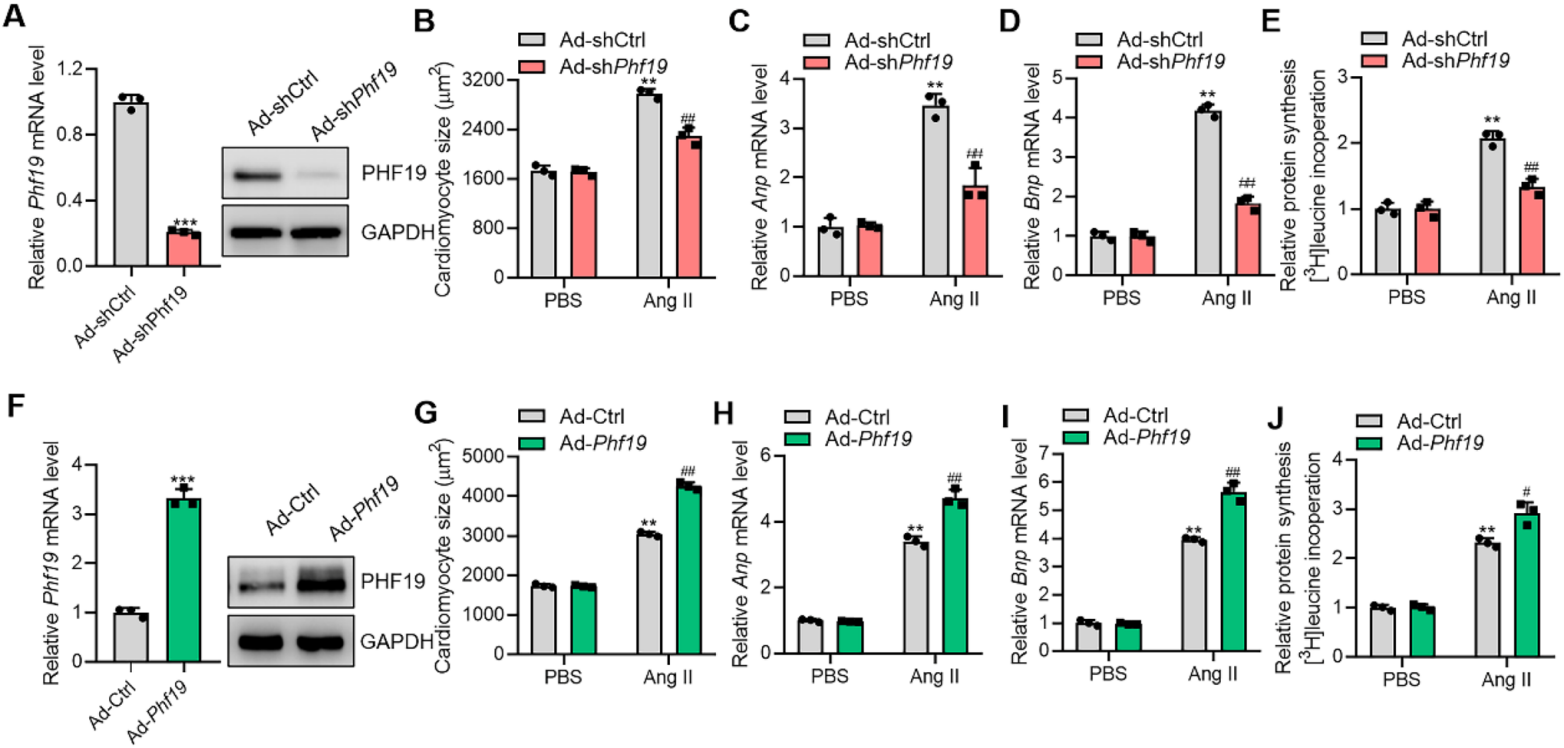
PHF19 promotes cardiomyocytes hypertrophy in vitro. **a** Knockdown of *Phf19* expression in cardiomyocytes. Neonatal rat cardiomyocytes were infected with adenovirus carrying shRNA targeting *Phf19* (Ad-sh*Phf19*) or control shRNA (Ad-shCtrl) (*n* = 3). Data are shown as mean ± SD. ***P < 0.001 versus Ad-shCtrl. **b–d** Knockdown of *Phf19* represses cardiomyocyte hypertrophy (*n* = 3). Neonatal rat cardiomyocytes were infected with Ad-*shPhf19* or Ad-shCtrl for 24 h, and then treated with angiotensin II (Ang II, 1 μM) for 48 h to induce hypertrophy. **b** Quantification of cardiomyocyte size. **c** mRNA level of *atrial natriuretic peptide (Anp)*. **d** mRNA level of *brain natriuretic peptide (Bnp)*. Data are shown as mean ± SD. **P < 0.01 versus Ad-shCtrl + PBS, ##*P* < 0.01 versus Ad-shCtrl + Ang II. **d** Knockdown of *Phf19* represses protein synthesis in cardiomyocytes (*n* = 3). Protein synthesis was monitored with the incorporation of [^3^H]-leucine. Data are shown as mean ± SD. **P < 0.01 versus shCtrl + PBS, ##*P* < 0.01 versus Ad-shCtrl + Ang II. **e** Overexpression of *Phf19* expression in cardiomyocytes (*n* = 3). Neonatal rat cardiomyocytes were infected with adenovirus carrying rat *Phf19* (Ad-*Phf19*) or control adenovirus (Ad-Ctrl). Data are shown as mean ± SD. ***P < 0.001 versus Ad-Ctrl. **f–h** Overexpression of *Phf19* promotes cardiomyocyte hypertrophy (*n* = 3). Neonatal rat cardiomyocytes were infected with Ad-*Phf19* or Ad-Ctrl for 24 h, and then treated with Ang II (1 μM) for 48 h to induce hypertrophy. **f** Overexpression of *Phf19* expression in cardiomyocytes. **g** Quantification of cardiomyocyte size. **h** mRNA level of *Anp*. **i** mRNA level of *Bnp*. Data are shown as mean ± SD. **P < 0.01 versus Ad-Ctrl + PBS, ##*P* < 0.01 *vs.* Ad-Ctrl + Ang II. **j** Overexpression of *Phf19* promotes protein synthesis in cardiomyocytes. Protein synthesis was monitored with the incorporation of [^3^H]-leucine. Data are shown as mean ± SD. **P < 0.01 versus Ad-Ctrl + PBS, ##*P* < 0.01 versus Ad-Ctrl + Ang II

Firstly, we generated adenovirus to knock down *Phf19* in neonatal rat cardiomyocytes to investigate the roles of PHF19 in cardiomyocyte hypertrophy. Adenovirus-mediated shRNA significantly silenced the expression of *Phf19* in cardiomyocytes (Fig. 1a). Next, cardiomyocyte hypertrophy was induced by treatment with Ang II. Ang II significantly increased the cardiomyocyte size and promoted the expression of hypertrophy-associated *Anp* and *Bnp* genes (Fig. 1b and c, Supplementary Figure 1). Thus, *Phf19* knockdown remarkedly repressed cardiomyocyte hypertrophy. Protein synthesis is a crucial feature of cardiomyocyte hypertrophy [7]. We observed that *Phf19* knockdown significantly reduced the increase in protein synthesis level induced by Ang II (Fig. 1d).

We also investigated whether Phf19 overexpression could promote cardiomyocyte hypertrophy by overexpressing rat *Phf19* in cardiomyocytes with adenovirus (Fig. 1e). *Phf19* overexpression alone was unable to induce hypertrophic growth of cardiomyocytes. However, *Phf19* overexpression significantly promoted Ang II-induced increase in cardiomyocyte size, expression of hypertrophic fetal genes, and protein synthesis (Fig. 1f–h, Supplementary Figure 1). Therefore, these findings demonstrated that PFH19 enhanced the hypertrophic response to stress stimuli (AngII) in vitro.

### PHF19 Promotes Cardiac Hypertrophy In Vivo

**Fig. 2:**
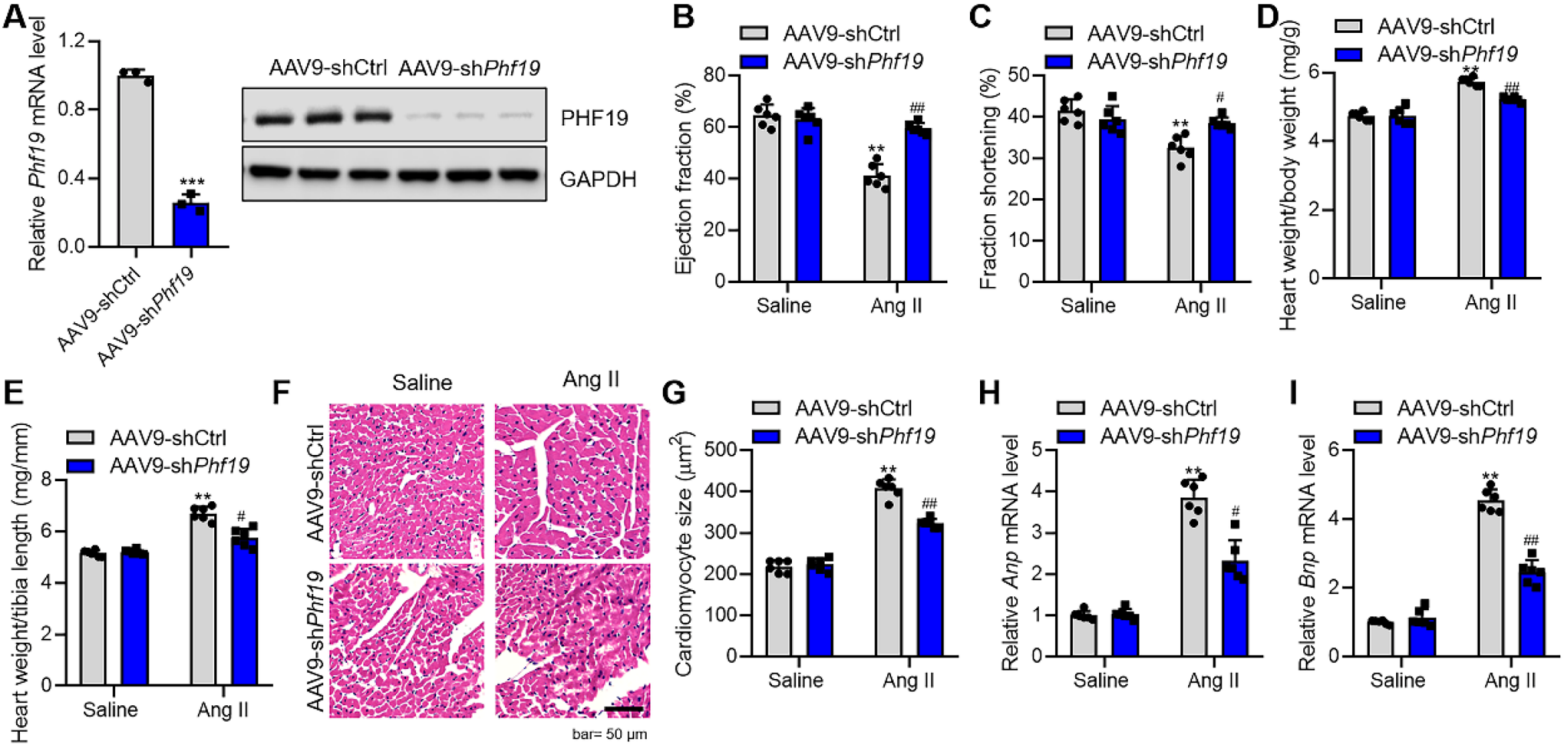
PHF19 promotes cardiac hypertrophy in vivo**. a** *Phf19* knockdown in cardiac tissues in vivo. Neonatal mice were treated with a single dose of adeno-associated virus 9 (AAV9) carrying shCtrl (AAV9-shCtrl) or sh*Phf19* (AAV9-sh*Phf19*). Data are shown as mean ± SD. *n* = 5 in each group. ***P < 0.001 versus AAV9-shCtrl. **b–c** *Phf19* knockdown represses Ang II-induced decline in cardiac function. Mice with/without Phf19 knockdown were subjected to Ang II treatment for four weeks, and then fraction shortening and ejection fraction were analyzed. Data are shown as mean ± SD. *n* = 6 in each group. **P < 0.01 versus AAV9-shCtrl + Saline, ##*P* < 0.01 versus AAV9-shCtrl + Ang II. **d–e** *Phf19* knockdown represses Ang II-induced increase in heart weight. Heart weight-to-body weight ratio (**d**) and heart weight to tibia length (**e**) are shown. Data are shown as mean ± SD. *n* = 6 in each group. **P < 0.01 versus AAV9-shCtrl + Saline, ##*P* < 0.01 versus AAV9-shCtrl + Ang II. **f–g** *Phf19* knockdown represses Ang II-induced increase in cardiomyocyte size. Representative images (**f**) and quantitative results (**g**) are shown. Data are shown as mean ± SD. *n* = 6 in each group. **P < 0.01 versus AAV9-shCtrl + Saline, ##*P* < 0.01 versus AAV9-shCtrl + Ang II. **h–i** *Phf19* knockdown represses Ang II-induced increase in expression of hypertrophic fetal genes. The expression of *Anp* (**h**) and *Bnp* (**i**) were analyzed by qRT-PCR. *n* = 6 in each group. Data are shown as mean ± SD. **P < 0.01 versus AAV9-shCtrl + Saline, ##*P* < 0.01 versus AAV9-shCtrl + Ang II

Next, we tested the functional importance of PHF19 in cardiac hypertrophy in vivo. To this end, we silenced the expression of *Phf19* in mouse hearts with adeno-associated virus 9-mediated shRNA (AAV9-sh*Phf19*). Neonatal mice were injected with a single dose of AAV9-shCtrl or AAV9-sh*Phf19*, and the knockdown efficiency was tested eight weeks later. Compared with the control AAV9-shCtrl, AAV9-sh*Phf19* reduced the expression level of *Phf19* in mouse hearts significantly (Fig. 2a). Mice received AAV9-shCtrl or AAV9-sh*Phf19* were chronically infused with Ang II for four weeks to induce pathological cardiac hypertrophy. Ang II treatment caused declines in cardiac function parameters, fraction shortening, and ejection fraction. Besides, Ang II treatment increased heart weight, cardiomyocyte size, and the expression of hypertrophic fetal genes *Anp* and *Bnp*. Interestingly, we observed that *Phf19* knockdown repressed the decline in cardiac function in mice with cardiac hypertrophy (Fig. 2b and c). In addition, *Phf19* knockdown repressed Ang II-induced increase in heart weight, cardiomyocyte size, and hypertrophic fetal genes (Fig. 2d-i). However, we did not observe the effects of PHF19 on heart rate and blood pressure (Supplementary Figure 2). Therefore, these findings demonstrated that *Phf19* knockdown inhibited cardiac hypertrophy in vivo*.*

### SIRT2 is Involved in the Function of PHF19

**Fig. 3:**
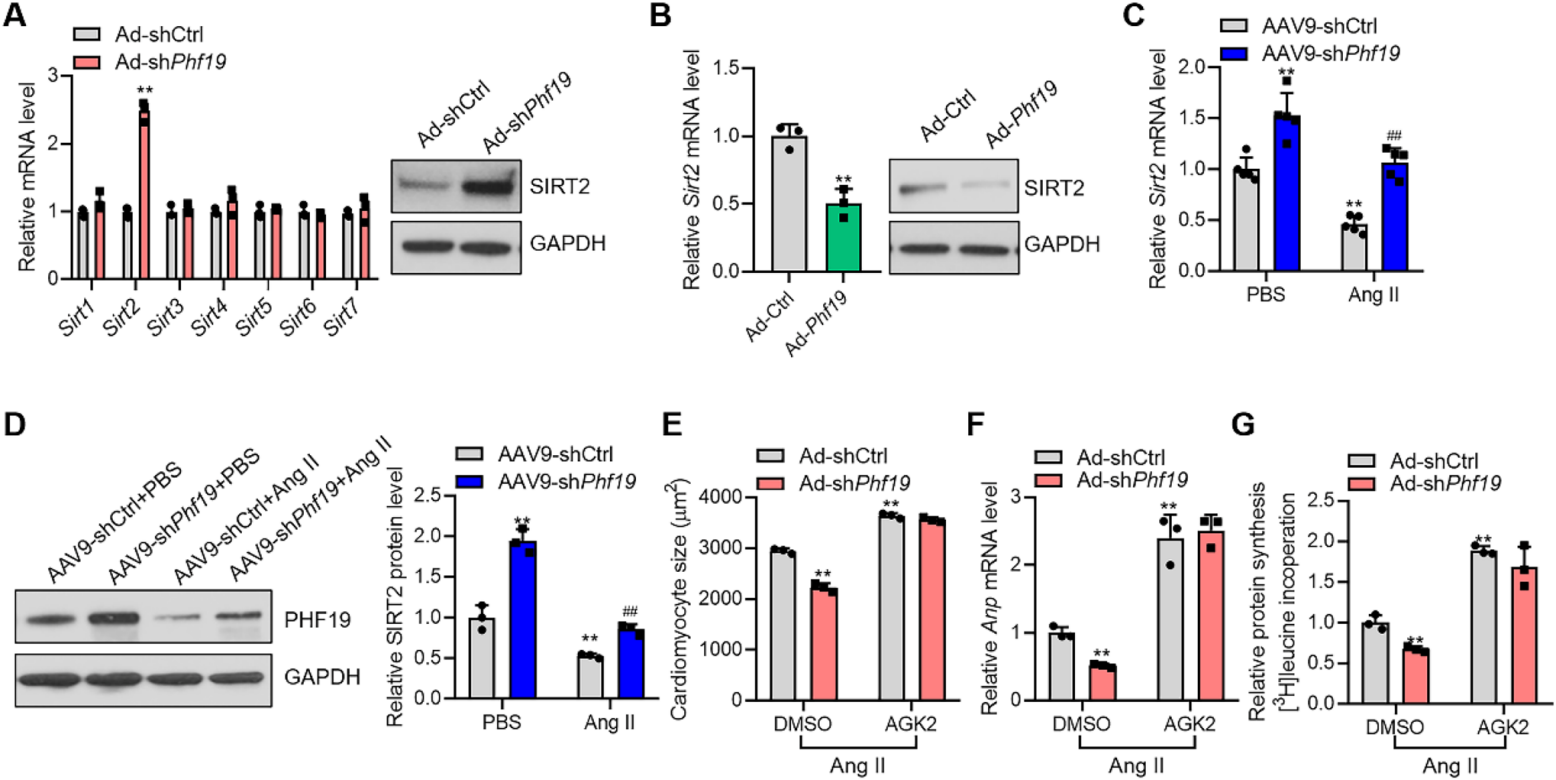
PHF19 regulates the expression of SIRT2 in cardiomyocytes and heart tissues.**a** Expression of Sirt1-7 in cardiomyocytes (*n* = 3). Neonatal rat cardiomyocytes were infected with Ad-shCtrl or Ad-sh*Phf19* for 24 h, and then the cells were treated with Ang II for additional 48 h. Data are shown as mean ± SD. **P < 0.01 versus Ad-shCtrl. **b** *Phf19* overexpression repressed the expression of *Sirt2* in cardiomyocytes (*n* = 3). Neonatal rat cardiomyocytes were infected with Ad-Ctrl or Ad-*Phf19* for 24 h, and then the cells were treated with Ang II for additional 48 h. Data are shown as mean ± SD. **P < 0.01 versus Ad-Ctrl. **c** *Phf19* knockdown repressed Ang II-induced downregulation of *Sirt2* (*n* = 3). Mice with/without *Phf19* knockdown were subjected to Ang II treatment for four weeks. Data are shown as mean ± SD. **P < 0.01 versus AAV9-shCtrl, ##*P* < 0.01 versus AAV9-shCtrl + Ang II. **d-f** SIRT2 was involved in *Phf19* function in cardiomyocyte hypertrophy (*n* = 3). Neonatal rat cardiomyocytes were infected with Ad-sh*Phf19* or Ad-shCtrl for 24 h, and then treated with angiotensin II (Ang II, 1 μM) for 48 h to induce hypertrophy in the presence/absence of SIRT2 inhibitor AGK2 (10 μM) or vehicle DMSO. **d** Expression of *Phf19* protein level. **e** Quantification of cardiomyocyte size. **f** mRNA level of *Anp*. **g** Protein synthesis was monitored by the incorporation of [^3^H]-leucine. Data are shown as mean ± SD. **P < 0.01 versus Ad-shCtrl + DMSO

Our findings demonstrated that PHF19 promoted cardiomyocyte hypertrophy in vitro and cardiac hypertrophy in vivo. Then, we explored the potential mechanism underlying PHF19 function during cardiomyocyte hypertrophy. Sirtuins are class III histone deacetylases, which play significant roles in cardiac hypertrophy [26]. We first tested the effects of *Phf19* on the expression of the members of Sirtuins. We observed that *Phf19* knockdown significantly upregulated the expression of *Sirt2* but not the other members of the Sirtuins, in rat cardiomyocytes (Fig. 3a). By contrast, *Phf19* overexpression reduced the expression of *Sirt2* in cardiomyocytes (Fig. 3b). During cardiac hypertrophy, the level of *Sirt2* was decreased in the heart tissues. Interestingly, *Phf19* knockdown increased the expression of *Sirt2* in heart tissues (Fig. 3c and d).

Next, we tested whether the upregulation of *Sirt2* contributed to the anti-hypertrophic roles of *Phf19* shRNA. To this end, SIRT2 was inhibited with a selective inhibitor AGK2 in cardiomyocytes. In Ang II-induced hypertrophic cardiomyocytes, AGK2 treatment significantly promoted hypertrophic growth of cardiomyocytes and repressed the expression of hypertrophic fetal genes and protein synthesis. Significantly, AGK2 treatment blocked the effects of *Phf19* knockdown on cardiomyocyte hypertrophy and protein synthesis (Fig. 3e-g, Supplementary Figure 3). Taken together, *Phf19* repressed *Sirt2* expression, which partially contributed to the function of *Phf19* during cardiomyocyte hypertrophy.

### PHF19 Represses SIRT2 Expression Epigenetically

**Fig. 4:**
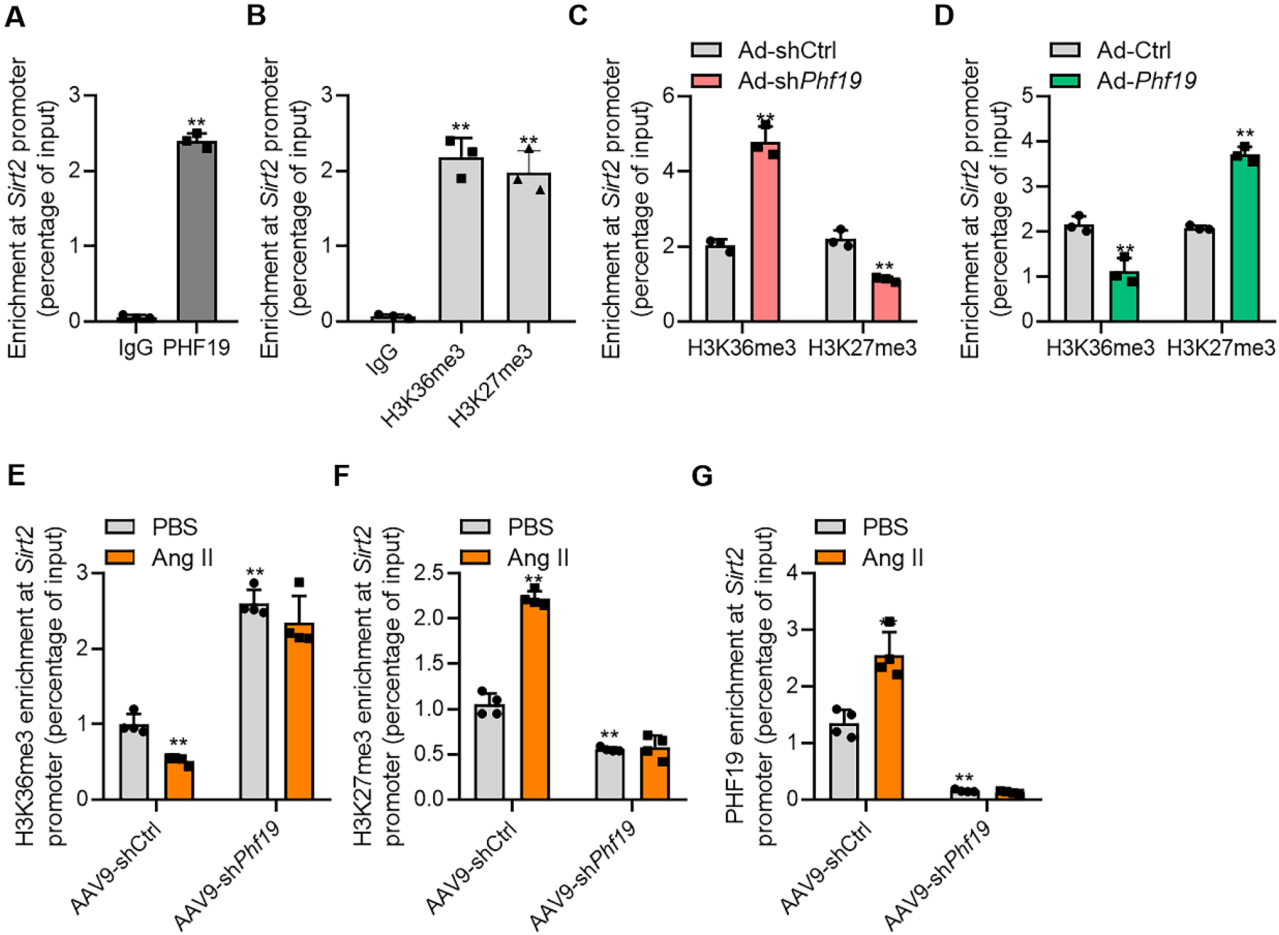
*Phf19* epigenetically regulates SIRT2 expression in cardiomyocytes**. a** PHF19 enrichment at *Sirt2* promoter in rat cardiomyocytes (*n* = 3). Chromatin immunoprecipitation (ChIP) was performed with anti-IgG or anti-PHF19 antibodies with lysis from rat cardiomyocytes, followed by qRT-PCR analysis. Data are shown as mean ± SD. **P < 0.01 versus IgG. **b** H3K27me3 and H3K36me3 enrichment at *Sirt2* promoter (*n* = 3). ChIP was performed with anti-IgG, anti-H3K27me3, or anti-H3K36me3 antibodies with lysis from rat cardiomyocytes, followed by qRT-PCR analysis. Data are shown as mean ± SD. **P < 0.01 versus IgG. **c** *Phf19* knockdown repressed H3K27me3 enrichment and increased H3K36me3 enrichment at *Sirt2* promoter in rat cardiomyocytes (*n* = 3). Data are shown as mean ± SD. **P < 0.01 versus Ad-shCtrl. **d** PHF19 overexpression increased H3K27me3 enrichment and inhibited H3K36me3 enrichment at *Sirt2* promoter in rat cardiomyocytes (*n* = 3). Data are shown as mean ± SD. **P < 0.01 versus Ad-Ctrl. **e** *Phf19* knockdown repressed Ang II-induced decline in H3K36me3 enrichment at *Sirt2* promoter in mouse hearts (*n* = 4). **P < 0.01 versus AAV9-shCtrl + PBS. **f** *Phf19* knockdown repressed Ang II-induced increase in H3K27me3 enrichment at *Sirt2* promoter in mouse hearts (*n* = 4). Data are shown as mean ± SD. **P < 0.01 versus AAV9-shCtrl + PBS. **g** *Phf19* knockdown repressed Ang II-induced increase in PHF19 enrichment at Sirt2 promoter in mouse hearts (*n* = 4). Data are shown as mean ± SD. **P < 0.01 versus AAV9-shCtrl + PBS

Then we investigated whether PHF19 regulated SIRT2 expression epigenetically. We performed a chromatin immunoprecipitation (ChIP) assay. We detected the enrichment of PHF19, H3K36me3, and H3K27me3 at the promoter of the *Sirt2* gene in cardiomyocytes (Fig. 4a and b). *Phf19* knockdown increased H3K36me3 while reduced H3K27me3 enrichment at *Sirt2* promoter (Fig. 4c). By contrast, *Phf19* overexpression significantly reduced H3K36me3 but increased H3K27me3 enrichment at the *Sirt2* promoter (Fig. 4d). During cardiac hypertrophy, Ang II treatment reduced the enrichment of H3K36me3 at *Sirt2* promoter in heart tissues, which was blocked by *Phf19* knockdown (Fig. 4e). The effects of Ang II on H3K27me3 enrichment at the *Sirt2* promoter were also blocked by the *Phf19* knockdown in cardiac tissues (Fig. 4f). In addition, the enrichment of H3K36me3 and H3K27me3 at *Sirt2* promoter was accomplished with PHF19 enrichment at *Sirt2* promoter (Fig. 4g). Furthermore, we tested whether PHF19 regulates histone modification via H3K36me3 demethylase NO66 [13]. However, we did not observe the effects of PHF19 on NO66 expression in cardiomyocytes (Supplementary Figure 4A). We also tested the enrichment of PHF19 on the promoters of other Sirtuins. The results showed that PHF19 did not bound to the promoters of other Sirtuins (Supplementary Figure 4B). Collectively, these findings demonstrated that PHF19 epigenetically regulated the expression of Sirt2 in cardiomyocyte hypertrophy.

### PHF19 Expression in Human Hypertrophic Hearts

**Fig. 5:**
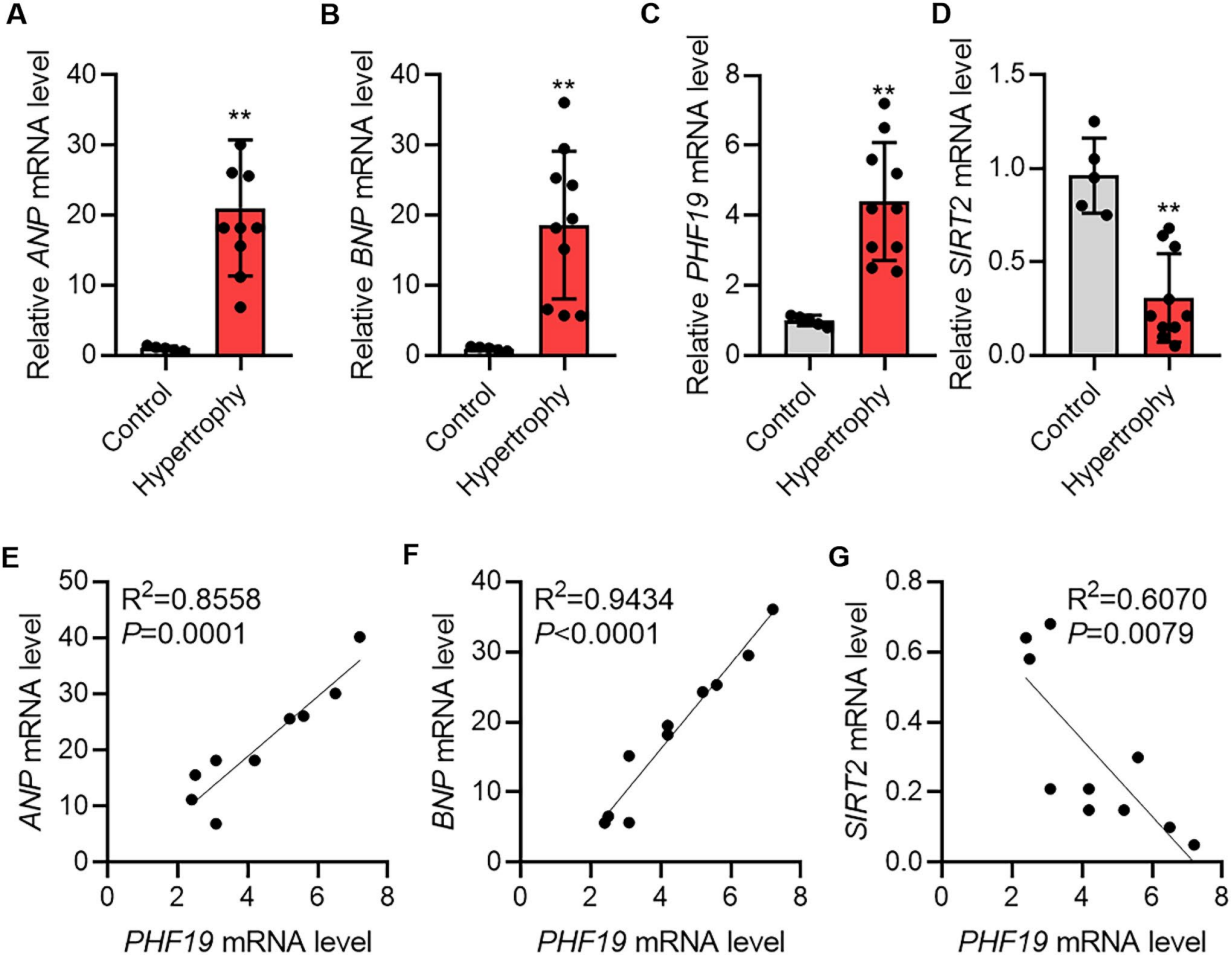
PHF19 and SIRT2 expression during human and mouse cardiac hypertrophy. **a–d** qRT-PCR showing the expression of *PHF19* (**a**), *ANP* (**b**), *BNP* (**c**), and *SIRT2* (**d**) in control (*n* = 5) and hypertrophic (*n* = 10) human hearts. Data are shown as mean ± SD. **P < 0.01 versus control. **e** Spearman correlation analysis showing *PHF19* expression was positively correlated with *ANP* expression in human hypertrophic hearts. **f** Spearman correlation analysis showing *PHF19* expression was positively correlated with *BNP* expression in human hypertrophic hearts. **g** Spearman correlation analysis showing *PHF19* expression was negatively correlated with *SIRT2* expression in human hypertrophic hearts

Finally, we analyzed the expression of PHF19 and SIRT2 in human hypertrophic hearts. The expression of *ANP* and *BNP* was significantly upregulated in hypertrophic hearts compared with the controls (Fig. 5a and d). We also observed the upregulation of *PHF19* and downregulation of *SIRT2* in human hypertrophic hearts (Fig. 5c and d). To determine the correlation of *PHF19* and other genes, Spearman correlation analysis was performed. The results showed that *PHF19* was positively correlated with the expression level of *ANP* and *BNP* (Fig. 5e and f). By contrast, *PHF19* was negatively correlated with the expression of *SIRT2* in human hypertrophic hearts (Fig. 5g). Collectively, these results revealed the upregulation of PHF19 in hypertrophic hearts and the correlation with hypertrophic fetal genes and SIRT2.

## Discussion

**Fig. 6:**
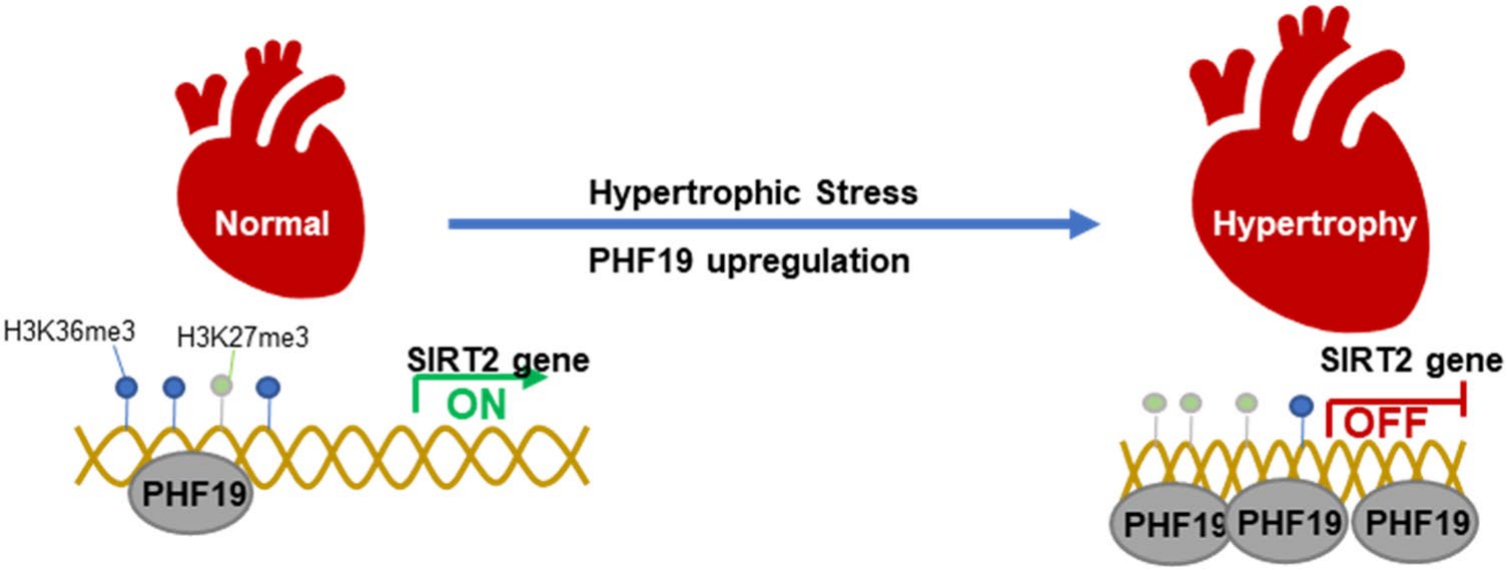
Graph showing mechanism of PHF19-SIRT2 axis in cardiac hypertrophy**.** Hypertrophic stress induces overexpression of PHF19, which binds SIRT2 promoter and leads to increased H3K36me3 enrichment and decreased H3K27me3 enrichment on SIRT2 promoter to repress the expression of the anti-hypertrophic factor SIRT2, and subsequently results in cardiac hypertrophy

In the present study, we provided evidence that PHF19 promotes cardiac hypertrophy via epigenetically repression SIRT2 expression. With *loss-of-function* and *gain-of-function* strategies, we demonstrated that PHF19 promoted cardiomyocyte hypertrophy and protein synthesis in vitro*.* We also observed that PHF19 knockdown with AAV9-mediated shRNA repressed pathological cardiac hypertrophy in vivo*.* Mechanism study identified SIRT2 as a downstream target for PHF19, and SIRT2 downregulation was critically essential for the pro-hypertrophic roles of PHF19. PHF19 regulated the switch of the enrichment of H3K36me3 and H3K27me3 at the promoter of SIRT2 to repress the expression of SIRT2 (Fig. 6). Finally, we observed the overexpression of PHF19 and downregulation of SIRT2 in human hypertrophic hearts.

Accumulating studies have supported the notion that epigenetic regulators critically participate in the development of cardiac hypertrophy and heart failure. JMJD2A was overexpressed in hypertrophic heart tissues and promoted the development of cardiac hypertrophy via regulating H3K9me3 [9]. The histone demethylase JMJD1C regulated H3K27me to repress CAMKK2-AMPK signaling and promote cardiac hypertrophy [10]. Besides, NSD2 promoted ventricular remodeling mediated by the regulation of H3K36me2 [27]. However, how these histone methylations cooperate to regulate cardiac hypertrophy remains unknown. PHF19 is a subunit of the polycomb repressive complex 2 (PRC2). The interaction of H3K36me2 and H3K36me3 with PHF19 is essential for PRC2 complex activity and proper gene repression [28]. PHF19 is associated with the H3K36me3 demethylase NO66, leading to PRC2-mediated H3K27me3, loss of H3K36me3, and transcriptional silencing [13]. The critical roles of PHF19 were observed in stem cell maintenance and differentiation [13, 14, 17, 28], cancer biology [15, 16], reprogramming of T cells [29], and rheumatoid arthritis [30]. But the potential roles of PHF19 in the cardiovascular system remain unknown.

In this study, we observed the overexpression of PHF19 in human hypertrophic hearts, which was correlated with the overexpression of hypertrophy-related fetal genes *ANP* and *BNP.* Our *loss-of-function* and *gain-of-function* experiments demonstrated the critical pro-hypertrophic roles of PHF19 in cardiomyocytes. Of importance, we demonstrated that PHF19 promoted stress-induced cardiac hypertrophy and facilitated the decline in fraction shortening and ejection fraction. Besides, we observed that PHF19 promoted protein synthesis, a core mechanism underlying the hypertrophic growth of cardiomyocytes [7]. Overall, PHF19 acts as a pro-hypertrophic factor and may serve as a target for the treatment of cardiac hypertrophy.

Histone acetylation also participates in cardiac hypertrophy, and inhibition of HDACs was considered as a promising strategy for the treatment of cardiac hypertrophy [5]. The Sirtuin family is a core regulator for cardiac homeostasis. SIRT1 was critical for cardiac development via regulating core cardiac transcription factors, and mutations in SIRT1 lead to development defects [31, 32]. SIRT2 was reported to repress cardiac hypertrophy via diverse mechanisms, including activation of AMPK and repression of GSK3b and NFATc2 [8, 20, 21]. The mitochondrial SIRT3 and SIRT5 were cardiac protective factors that repress aging and pathological cardiac hypertrophy, whereas SIRT4 was reported as the only member as a pro-hypertrophic member [26, 33–35]. SIRT6 and SIRT7 were critical for heart development and repress cardiac hypertrophy via modulating histone acetylation and transcription factors [36]. Although the roles of Sirtuins have been identified in cardiac hypertrophy, the mechanisms underlying the regulation of Sirtuin expression during cardiac hypertrophy were not fully understood.

Here in this study, we observed that PHF19 repressed the expression of SIRT2 but not other members of Sirtuins. PHF19 bound the promoter of SIRT2 to modulate the enrichment of H3K36me3 and H3K27me3 at the SIRT2 promoter, subsequently leading to the suppression of SIRT2 expression. Based on previous literature [14, 17, 28], one of the mechanisms underlying PHF19-mediated SIRT2 repression is the switch of H3K36me3 and H3K27me3 at the SIRT2 promoter. PHF19 cooperated with PRC2, KDM2b/NO66 complex. PHF19 can bound H3K36me3 directly, which leads to a reduction of H3K36me3 by KDM2b/NO66. PHF19 also recruited PRC2, which increased the level of H3K27me3, the repressor of transcription. The increased H3K36me3 and increased H3K27me3 at the SIRT2 promoter tied the chromatin to reduce SIRT2 transcription. Our studies showed that the regulation of PHF19 on SIRT2 did not rely on NO66 expression change. Further studies are needed to determine whether PRC2 and NO66 are critically crucial for the PHF19-mediated repression of SIRT2.

The negative correlation of PHF19 and SIRT2 expressions was also observed in human hypertrophic hearts. We observed that SIRT2 was critically important for the functions of PHF19 in regulating protein synthesis and hypertrophic growth of cardiomyocytes. SIRT2 was a core deacetylase for histones, cytoplasmic and mitochondrial proteins. Thus, we identified the communication between protein methylation and acetylation within hypertrophic hearts, the imbalance of which may be one of the core mechanisms underlying cardiac hypertrophy.

In conclusion, we identified PHF19 as a pro-hypertrophy factor in pathological cardiac hypertrophy. PHF19 overexpression in the hearts leads to the imbalance of H3K36me3 and H3K27me3 to repress the expression of SIRT2, which is critical for the pro-hypertrophic function of PHF19. Therefore, PHF19 may serve as a target for the treatment of cardiac hypertrophy.

## Supplementary Information

Below is the link to the electronic supplementary material.

Supplementary material 1 (DOCX 446 kb)
