# Supplementary material for: PHD Finger Protein 19 Promotes Cardiac Hypertrophy via Epigenetically Regulating SIRT2
Source: Cardiovasc Toxicol. 2021 Feb 21;21(6):451–61. doi: 10.1007/s12012-021-09639-0 (PMC8076129; doi:10.1007/s12012-021-09639-0)
Supplement: Supplementary file 1 — Supplementary material 1 (DOCX 446 kb) [file 12012_2021_9639_MOESM1_ESM.docx]

**
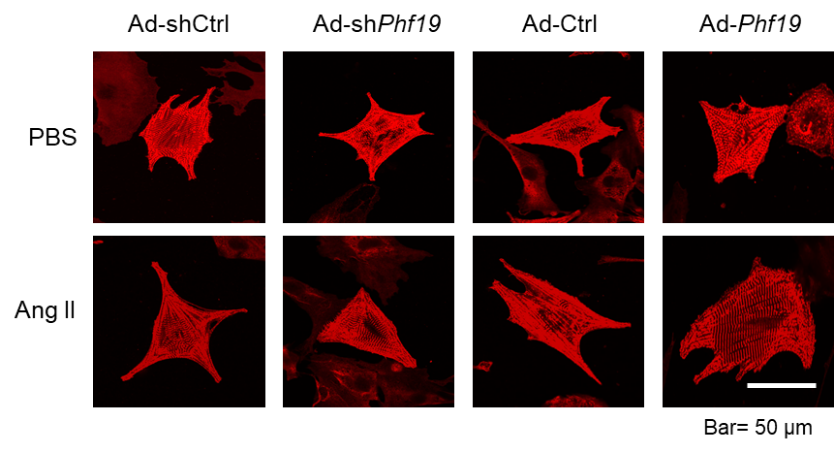
Supplementary data**


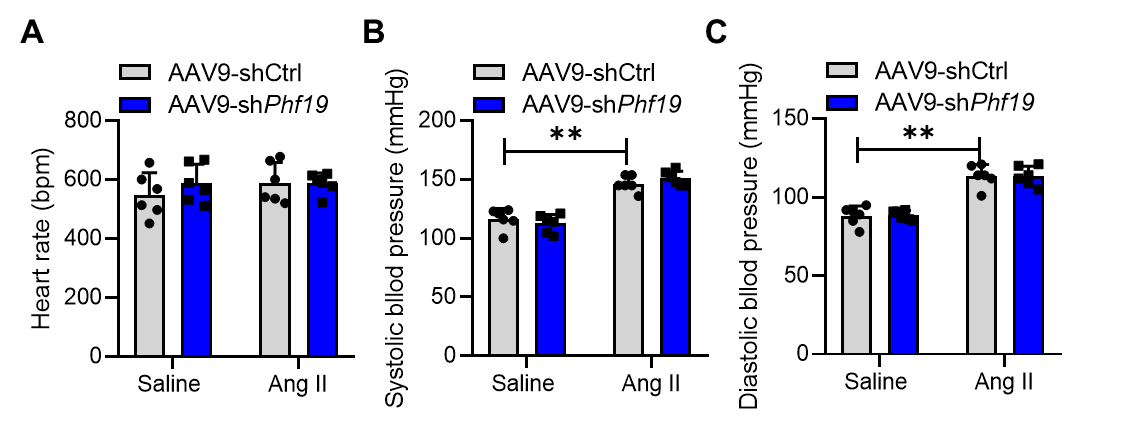
**Supplementary Figure 1. PHF19 regulates Ang II-induced cardiomyocyte hypertrophy.** Data related to Figure 1.

**
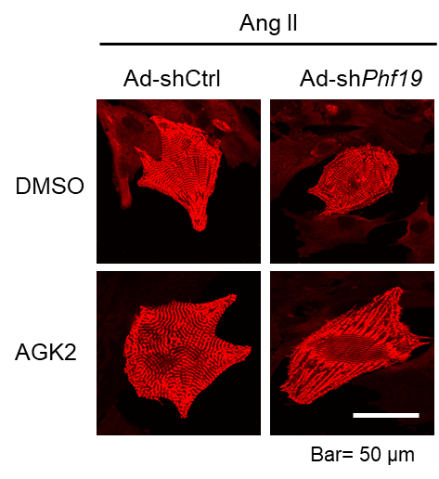
Supplementary Figure 2. PHF19 knockdown does not affect heart rate and blood pressure in mice.** Data related to Figure 2.


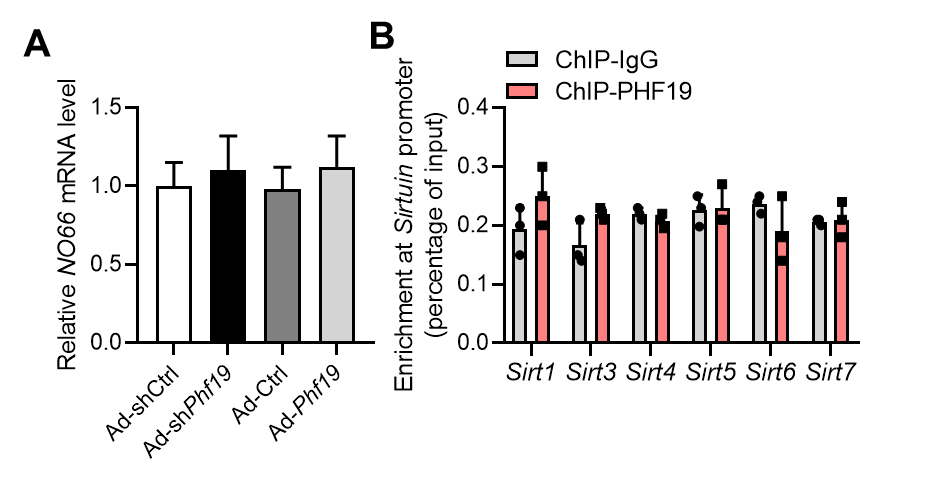
**Supplementary Figure 3. SIRT2 contributes to PHF19 function in regulating cardiomyocyte hypertrophy.** Data related to Figure 3.

**Supplementary Figure 4. Epigenetic regulation of Sirtuins.** Data related to Figure 4.
